# Insulin Therapy in Type 2 Diabetes Is Associated With Barriers to Activity and Worse Health Status: A Cross-Sectional Study in Primary Care

**DOI:** 10.3389/fendo.2021.573235

**Published:** 2021-03-10

**Authors:** Anne Meike Boels, Guy Rutten, Frits Cleveringa, Mariëlle van Avendonk, Rimke Vos

**Affiliations:** Julius Center for Health Sciences and Primary Care, University Medical Center Utrecht, Utrecht, Netherlands

**Keywords:** type 2 diabetes, insulin, oral antihyperglycemic agents, health status, psychosocial functioning

## Abstract

**Introduction:**

Many individuals with type 2 diabetes mellitus (T2DM) experience “psychological insulin resistance”. Consequently, it could be expected that insulin therapy may have negative effects on psychological outcomes and well-being. Therefore, this study compared health status and psychosocial functioning of individuals with T2DM using only oral antihyperglycemic agents (OHA) and on insulin therapy (with or without OHA).

**Materials and Methods:**

In this cross-sectional study, we used baseline data of a cluster randomized controlled trial conducted in 55 Dutch general practices in 2005. Health status was measured with the Short Form (SF)-36 (scale 0–100) and psychosocial functioning with the Diabetes Health Profile (DHP, scale 0-100). To handle missing data, we performed multiple imputation. We used linear mixed models with random intercepts per general practice to correct for clustering at practice level and to control for confounding.

**Results:**

In total, 2,794 participants were included in the analysis, their mean age was 65.8 years and 50.8% were women. Insulin-users (n = 212) had a longer duration of T2DM (11.0 versus 5.6 years) and more complications. After correcting for confounders and multiple comparisons, insulin-users reported significantly worse outcomes on vitality (SF-36, adjusted difference -5.7, p=0.033), general health (SF-36, adjusted difference -4.8, p=0.043), barriers to activity (DHP, adjusted difference -7.2, p<0.001), and psychological distress (DHP, adjusted difference -3.7, p=0.004), all on a 0-100 scale.

**Discussion:**

While previous studies showed similar or better health status in people with type 2 diabetes receiving insulin therapy, we found that vitality, general health and barriers to activity were worse in those on insulin therapy. Although the causality of this association cannot be established, our findings add to the discussion on the effects of insulin treatment on patient-reported outcomes in daily practice.

## Introduction

Insulin therapy may be essential for many patients during the course of type 2 diabetes mellitus (T2DM) (1). While insulin has greater efficacy to lower glycated hemoglobin (HbA1c) compared to oral antihyperglycemic agents (OHA) (2), still many individuals with T2DM are reluctant to start insulin therapy. This “psychological insulin resistance” includes fear of hypoglycemia and weight gain, fear for injections and feelings of guilt and failure (3–8). Consequently, it could be expected that insulin therapy may have negative effects on psychological outcomes and well-being.

Nevertheless, studies in patients who had recently initiated insulin therapy, showed either positive (9–15) or no effects (16–22) on health status and well-being. In contrast, studies in patients who had been using insulin for a longer period found a negative association between insulin therapy and health status (23–25). A recent observational longitudinal study showed that at baseline and during follow-up, individuals with stable insulin therapy had the lowest health status (physical component scale); those who initiated insulin therapy had an unaltered health status (26). These studies however, had methodological limitations: the number of insulin-users was small (25), the selection of confounders was data-driven (24), or there was no adjustment for potential confounders (26).

The aim of this study was to compare health status and psychosocial functioning between individuals with T2DM using only OHA and those using insulin therapy with or without OHA in a real-life context in a mixture of individuals who recently initiated insulin and those using insulin for a longer period of time. Our study adds to the body of existing knowledge and in our opinion deals with methodological limitation of prior studies.

## Materials and Methods

### Study Design, Setting, and Population

In this observational cross-sectional study we used baseline data from a cluster randomized controlled trial (RCT) conducted in 55 general practices in the Netherlands (27). It investigated the effects of a diabetes care protocol (27). Participants were recruited in 2005. All registered T2DM patients were eligible to participate, but those with a short life expectancy, unable to visit the general practice, receiving diabetes treatment at hospital outpatient clinics (secondary care), or those refusing to participate were excluded. For the purposes of the current study, we also excluded T2DM patients who did not use blood glucose lowering medication, but only had a lifestyle advice. The University Medical Centre Utrecht ethics committee approved the original study; patients provided written consent.

### Data Collection

The following participants’ characteristics were registered on electronic patient files: age, sex, diabetes duration, systolic blood pressure (SBP), body mass index (BMI), HbA1c, lipid profile, level of education, ethnicity, microvascular complications, and macrovascular complications. Systolic blood pressure and body mass index (BMI) were assessed by the practice nurse. HbA1c and lipid profile were measured in local laboratories. Level of education was categorized into low (primary school, pre-vocational education), intermediate (higher general continued education, preparatory scholarly education, middle-level applied education), or high (university of applied science, research university). Ethnicity was categorized into Western-European or other. Microvascular complications were defined as presence of retinopathy (assessed by fundus screening), neuropathy (assessed by feet examination), and/or presence of nephropathy (urine albumin to creatinine ratio >2.5 mg/mmol for men and >3.5 mg/mmol for women, and/or an estimated glomerular filtration rate < 60 ml/min/1.73m2). Macrovascular complications were classified as present if angina pectoris, myocardial infarction, or cerebral infarction was recorded. Medication use was recorded by Anatomical Therapeutic Chemical codes. Insulin use was also identified based on ATC-codes (28).

### Questionnaires

Practice nurses handed out questionnaires to the participants, who completed these at home and returned them in a postage paid envelope to the research center. When the questionnaires were not returned within three months, participants received a reminder. For the current study, we used the Short Form-36 (SF-36) and the Diabetes Health Profile (DHP-1). Participants completed the SF-36 and the DHP-) before the intervention from the original cluster RCT took place.

The SF-36 is a 36-item questionnaire which assesses health status, encompassing nine dimensions: physical functioning (10 items), limitations due to physical difficulties (role physical, four items), bodily pain (two items), general health (six items), vitality (four items), social functioning (two items), limitations due to emotional difficulties (role emotional, three items), mental health (five items), and health change (one item). Items are rated on a 2–6-point Likert scales. For each of these dimensions, scores were transformed to a scale ranging from 0 to 100, with higher scores indicating better health (29).

The DHP-1 is a 32-item questionnaire which assesses the impact of diabetes on psychosocial functioning. It comprises three dimensions: psychological distress (14 items, e.g., dysphoric mood, feelings of hopelessness), barriers to activity (13 items on activity restriction due to diabetes, e.g., avoiding going out when blood glucose is on the low side) and disinhibited eating (five items measuring response of emotional arousal and external food cues, e.g., lack of eating restraint). Items are rated on a 4-point Likert scale ranging from 0 (“never” or “not at all”) to 3 (“very often” or “very much”) (30). For each dimension, scores were transformed to a scale ranging from 0 to 100, where 100 indicates no dysfunction.

### Analysis

Since missing data may lead to imprecision and biased results, we performed multiple imputation to handle missing data. Characteristics of participants with any missing value on the SF-36 or DHP-1, and those with complete data are shown in **Supplementary File 1**, suggesting data are missing at random. Under the missing at random assumption, we created 10 imputed datasets with 70 iterations (see **Supplementary File 2** for the full imputation strategy). Rubin’s rule was used to combine the multiple imputed estimates (31).

Differences between participants using only OHA and those using insulin (with or without OHA) were analyzed with t-test for continuous variables and χ^2^ test for categorical variables. To investigate the patient-reported outcomes (SF-36 and DHP) we used linear mixed models with random intercepts per general practice to correct for clustering at practice level. First, a univariable analysis was performed in which only the patient-reported outcome and insulin use were taken into account. Afterwards we conducted multivariable analyses corrected for confounders. We pre-specified the following confounders: sex, age, diabetes duration, ethnicity, level of education, microvascular and macrovascular complications, BMI, SBP, HbA1c, and LDL-cholesterol. Sex, ethnicity, microvascular, and macrovascular complications were entered as binary variables; level of education as a categorial variable. Age, diabetes duration, BMI, SBP, HbA1c, and LDL-cholesterol were entered as continuous variables. Assumptions of the models were assessed in each imputed dataset using residual analysis. A p-value <0.05 was considered to be statistically significant. The p-values from the multivariable analysis were corrected for multiple comparisons by the Holm-Bonferroni Sequential Correction method (32). We used RStudio version 1.0.143 for the statistical analyses and mice 3.3.0 package for multiple imputation (31).

## Results

**Figure 1:**
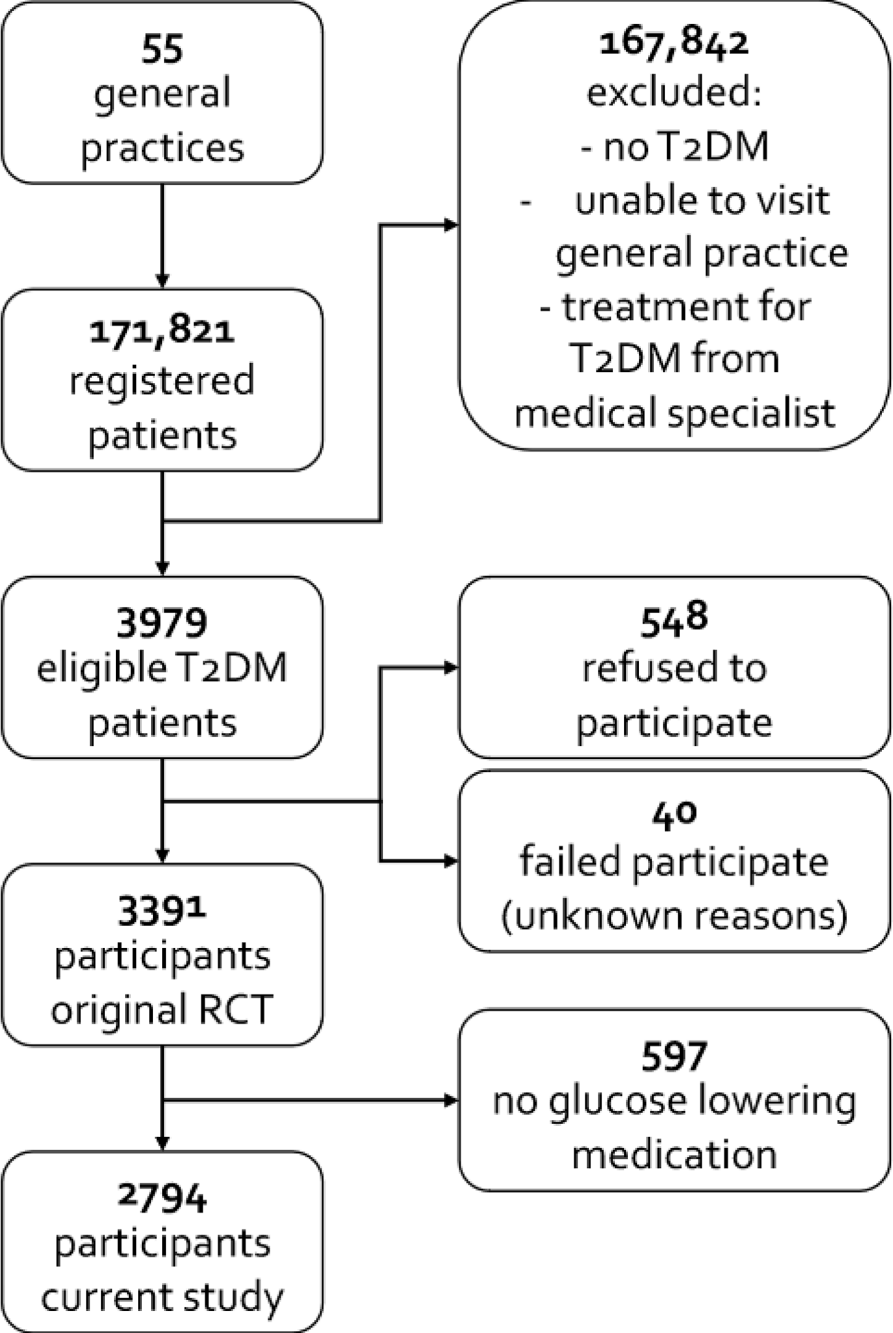
Flowchart. T2DM, type 2 diabetes mellitus.

Of the 3,979 eligible participants, 548 refused to participate and 40 failed to participate for unknown reasons. The final study population therefore consisted of 3,391 participants. Of these 3,391 participants, 597 did not use glucose lowering medication and were excluded (see **Figure 1**). The remaining 2,794 participants had 34.2% missing values with regard to the outcomes, distributed among 48.4% of the participants, and 6.4% missing values concerning the confounders, distributed among 52.7% of the participants.

The majority of the participants on insulin therapy (n = 212) also used OHA (59.4%); 86 out of 212 patients (40.6%) did not use OHA. **Table 1** shows that patients who received insulin therapy had a longer diabetes duration, were more often women, and had more microvascular and macrovascular complications. Glycemic control was worse and BMI was higher in insulin-treated patients. **Supplementary File 3** gives an overview of the types of insulin used by the study population. More than half of our population used pre-mixed insulin (see **Supplementary File 3**).

**Table 1:** Characteristics of type 2 diabetes patients treated in primary care using only oral antihyperglycemic agents or using insulin with or without oral antihyperglycemic agents.

	OHA only (n = 2582)	Insulin ± OHA (n = 212)	P-value
Sex: female (n (%))	1297 (50.2)	123 (58.0)	0.035
Age (years)	65.7 (11.1)	67.1 (11.1)	0.064
Duration diabetes (years)	5.6 (5.9)	11.0 (6.7)	<0.001
Ethnicity: western European (n (%))	2,399 (92.9)	201 (94.8)	0.321
Education (n (%))			0.060
Low	1,731 (67.0)	143 (67.5)	
Medium	624 (24.2)	55 (25.9)	
High	227 (8.8)	14 (6.6)	
Microvascular complications (n (%))	903 (35.0)	107 (50.5)	<0.001
Macrovascular complications (n (%))	441 (17.1)	49 (23.1)	0.033
Body mass index (kg/m^2^)	30.0 (5.3)	30.9 (5.4)	0.031
Systolic blood pressure (mmHg)	148.8 (21.4)	149.4 (21.6)	0.690
HbA1c (mmol/mol)	54.0 (12.8)	62.1 (12.9)	<0.001
HbA1c (%)	7.09 (1.17)	7.83 (1.18)	
LDL cholesterol (mmol/l)	2.72 (0.94)	2.63 (0.94)	0.167

All data are given in mean (SD), unless stated otherwise.

HbA1c, glycated haemoglobin; LDL, low-density lipoprotein; n, number; OHA, oral antihyperglycemic agents; SD, standard deviation.

**Table 2** shows the results of both the univariable and the multivariable linear mixed models. In the univariable analyses, before adjustment for confounding, individuals treated with insulin (with or without OHA) scored statistically significantly worse on nearly all SF-36 and DHP scales. In the univariable analyses, there were no associations between insulin use and health change (SF-36) and disinhibited eating (DHP). After adjustment for confounding, individuals treated with insulin (with or without OHA) reported statistically significantly worse outcomes on six scales of the SF-36 questionnaire: physical functioning, social functioning, role physical, mental health, vitality and general health. With regard to the DHP, patients treated with insulin (with or without OHA) scored statistically significantly lower on DHP barriers to activity and DHP psychological distress (i.e., more dysfunction). Residual analysis showed no deviation from distributional assumptions and no heteroscedasticity. After correcting for multiple comparisons, the associations between insulin use, and vitality and general health (SF-36), and those between insulin use and barriers to activity and psychological distress (DHP) remained statistically significant (**Table 2**).

**Table 2:** Patient reported outcomes of type 2 diabetes patients using only oral antihyperglycemic agents or using insulin with or without oral antihyperglycemic agents.

	OHA only (SD)(n = 2582)	Insulin ± OHA (SD)(n = 212)	Unadjusted difference (95% CI)	P-value	Adjusted difference (95% CI)*	P-value	Adjusted p-value**
**Short Form (SF)-36 (scale ranging from 0 to 100, with higher scores indicating better health)**							
Physical functioning	70.4 (37.0)	61.5 (33.3)	-8.9 (-13.4 to -4.3)	<0.001	-5.7 (-10.0 to -1.3)	0.011	0.076
Social functioning	82.4 (39.5)	75.7 (32.9)	-6.7 (-11.2 to -2.2)	0.003	-5.8 (-10.4 to -1.1)	0.016	0.095
Role physical	69.0 (56.6)	57.3 (52.2)	-11.7 (-19.0 to -4.5)	0.002	-8.6 (-15.8 to -1.3)	0.021	0.104
Role emotional	78.0 (55.0)	68.9 (53.3)	-9.2 (-16.7 to -1.7)	0.018	-6.4 (-14.4 to 1.7)	0.123	0.435
Mental health	75.7 (31.6)	70.2 (23.0)	-5.5 (-8.5 to -2.5)	<0.001	-4.5 (-7.8 to -1.3)	0.006	0.052
Vitality	62.4 (29.8)	55.0 (27.5)	-7.4 (-11.2 to -3.6)	<0.001	-5.7 (-9.5 to -2.0)	0.003	0.033
Bodily pain	78.4 (40.6)	72.8 (36.2)	-5.6 (-10.4 to -0.8)	0.024	-4.0 (-8.8 to 0.9)	0.109	0.435
General health	59.5 (33.1)	53.3 (24.5)	-6.2 (-9.4 to -2.9)	<0.001	-4.8 (-8.1 to -1.5)	0.005	0.043
Health change	50.2 (30.8)	48.9 (26.3)	-1.3 (-4.9 to 2.3)	0.477	0.3 (-3.4 to 3.9)	0.892	1.000
**Diabetes Health Profile (DHP) (scale ranging from 0 to 100, where 100 indicates no dysfunction)**							
Barriers to activity	86.8 (33.1)	78.6 (19.8)	-8.2 (-10.8 to -5.7)	<0.001	-7.2 (-9.9 to -4.6)	<0.001	<0.001
Psychological distress	87.9 (21.0)	84.1 (13.9)	-3.8 (-5.6 to -1.9)	<0.001	-3.7 (-5.8 to -1.7)	<0.001	0.004
Disinhibited eating	72.2 (30.2)	70.3 (28.2)	-1.9 (-5.7 to 1.9)	0.339	-1.0 (-4.9 to 3.0)	0.629	1.000

CI, confidence interval; n, number; OHA, oral antihyperglycemic agents; SD, standard deviation.

*Adjusted for: sex, age, diabetes duration, ethnicity, level of education, microvascular and macrovascular complications, BMI, systolic blood pressure, HbA1c, and LDL-cholesterol.

**P-values from multivariable analysis adjusted for multiple comparisons by the Holm-Bonferroni Sequential Correction method.

## Discussion

### Summary of Findings

Individuals with T2DM who use insulin reported worse vitality and general health, more psychological distress and more barriers to activity in comparison with patients using only OHA, independent of sex, age, diabetes duration, ethnicity, level of education, microvascular and macrovascular complications, BMI, systolic blood pressure, HbA1c, and LDL-cholesterol.

### Implications of Findings

With regard to the SF-36, the statistically significant differences can be considered clinically relevant, when the often suggested minimal important differences (MID) ranging from 3–5 points are taken into account (33). Taking the MIDs for the DHP-18 (developed from the DHP-1) into account, only the difference for barriers to activity was both statistically significant and clinically relevant (34).

Although we cannot ascertain a causal relationship between insulin use, and health status and psychosocial functioning, the findings of this study imply that it is important to make a well-considered decision about the initiation of insulin. Further, it is recommended to include patient-reported outcome measures in future trials comparing glucose lowering medication, insulin or different insulin regimens, keeping the duration of insulin therapy for the latter in mind.

### Comparison With Existing Literature

Previous studies with positive or neutral effects on patient-reported outcomes were conducted in the context of starting insulin therapy rather than continued use (9–22). Most studies were of observational nature – either cross-sectional or longitudinal (both retrospective and prospective studies). These observational studies always face the difficulty of dealing with confounding factors and questions about causal inference. For cross-sectional studies like ours, making causal inferences is even harder since there is no longitudinal aspect. An RCT deals with confounding factors – both measured and unmeasured confounding. Unfortunately, most RCTs have a short follow-up duration, which makes it impossible to make inferences on long term effects. Only one RCT specifically investigated quality of life after insulin initiation (11). The authors of this RCT randomized T2DM with poor glycemic control into one group with early insulin initiation and one group with adjustment of OHA. They found that quality of life improved in both the group of groups, but significantly more in the insulin group. The follow-up duration in this study was only 24 weeks. The association of the start of insulin therapy with improved health status and psychosocial functioning might be the effect of the diminishing symptoms of hyperglycemia. Our study was conducted in a different context, in a mixture of individuals who recently started using insulin and those using it for a longer period of time. We were unable to take duration of insulin therapy into account, but we adjusted for diabetes duration. Longer disease duration may be associated with a higher number and more serious diabetes-related complications and with longer insulin use. Multiple complications are important determinants of impaired health status, which underpins the importance of taking them into account (35). After adjustment for the occurrence of complications, most patient-reported outcomes remained worse in the insulin group, which makes our findings more robust.

Comparable to our study, two other studies conducted in the same context showed negative effects on perceived health status. A Dutch primary care-based study of 1,348 T2DM patients found that insulin therapy was associated with a worse health state (23). The other study found that individuals on insulin therapy with good metabolic control had a lower quality of life compared to those on OHA with poor metabolic control (25). As in our study, the duration of insulin therapy was unknown in these studies.

Two Australian cohort studies by the same authors studied the association between insulin use and health status cross-sectionally as well as longitudinally (both four years follow-up, in 1,290 (24) and 930 (26) T2DM patients). Both studies found that at baseline the insulin-treated individuals had a worse health status compared to non-insulin treated patients, and that the initiation of insulin therapy did not alter health status (subsamples of 38 (24) and 85 (26) patients). The most recent study also found that among those on stable insulin therapy, health status was lowest at all time-points during follow-up (26). The authors conclude that the burden of disease – diabetes duration, worse glycemic control, and higher number of complications – rather than insulin use determines health status (26). Interestingly, we adjusted for these factors and still found a lower health status among those using insulin.

### Strengths and Limitations

Our large sample size is a strength of the study. Due to the cross-sectional design, no causal associations between insulin use and patient-reported outcomes can be assumed. The participating practices were representative for primary care centers in the Netherlands, and the same applies to our study population. However the practices were self-selected, which might reflect special interest in improving diabetes care; extra emphasis on diabetes care might cause better results on patient-reported outcomes (27). Our participants were all treated in primary care. In the Netherlands, patients are referred to an internal medicine specialist or endocrinologist in secondary care when adequate glycemic control cannot be achieved or when problems occur that are beyond the scope of the primary care physician. Since in general, patients treated in secondary care have a higher disease burden, the results of our study may not be fully generalizable to patients treated in secondary care.

Since individuals on insulin therapy might have a different stage of disease and disease severity, we corrected for multiple confounders. Unfortunately, data on duration of insulin therapy were not available, while it could be an important effect modifier. We considered including an interaction term for diabetes duration * insulin use, as a proxy for insulin therapy duration. However, as this interaction term would not have been able to differentiate between an individual with a diabetes duration of ten years who started insulin 3 months ago, and an individual with a diabetes duration of ten years who started insulin 9 years ago, we decided this could lead to bias, and hence omitted this interaction. Moreover, we were unable to take type of insulin regiment into account. While type of insulin was registered, we could not differentiate between basal, mix of basal-prandial insulin schemes with sufficient certainty. This was unfortunate, since type of insulin regimen appears to influence quality of life.(Polonsky 2014) Also, novel OHA, e.g., SGLT2 inhibitors, and other agents, e.g., GLP-1 agonists, have emerged since the data were collected. These novel agents might influence the psychological well-being of individuals with T2DM. For example, semaglutide compared with insulin glargine statistically significantly improved the role-emotional and general health domains of the SF-36 but not on other SF-36 domains (36). Nevertheless, even nowadays millions of people all over the world start with insulin therapy instead of GLP-1 receptor agonists or SGLT2 inhibitors. This makes our current study relevant, despite the older data. Moreover, acceptability of insulin therapy may have changed too since the data were collected. We have not analyzed the outcomes for the three groups, i.e., (1) insulin only (2) OHA only and (3) insulin+OHA for two main reasons. In the Dutch Guideline for General Practitioners, as in many other (inter)national guidelines, it is advised to continue OHA when initiating insulin therapy. This means that the “insulin only group” is a group that either does not receive the appropriate therapy, or is a group for which the guideline is abandoned on purpose. In the latter case, reasons to do so are severe side effects from OHA, or chronic kidney disease. Comparing this group to, e.g., the “insulin+OHA group”, there are many unmeasured confounders for which we cannot correct with the available data. Functional decline, distress, and depression are strongly associated with exposure level. We cannot ignore the exposure level, even if it is a cross-sectional study. However, we decided not to use depression as a confounder, since it is even more likely that it is an intermediate factor in the causal pathway. Lastly, residual or unmeasured confounding might still be present.

### Conclusion

While shortly after insulin initiation health status may be uninfluenced or positively influenced, we found that in the real-life context, in a mixture of individuals who recently initiated insulin and those using insulin for a longer period of time, vitality, general health, and barriers to activity were worse in those on insulin therapy. Although the causality of this association has not been established, our findings once again stress the need to balance the beneficial effects of insulin therapy against the possible negative effects on patient-important outcomes in daily practice.

## Data Availability Statement

The data analyzed in this study is subject to the following licenses/restrictions: Data sharing upon request. Requests to access these datasets should be directed to r.c.vos@lumc.nl.

## Ethics Statement

The studies involving human participants were reviewed and approved by The University Medical Center Utrecht ethics committee. The patients/participants provided their written informed consent to participate in this study.

## Author Contributions

GR and MA designed the current study. Data were collected by FC and analyzed by AMB. Both MA and AMB wrote the manuscript. All authors contributed to the interpretation of data and to the discussion, reviewed and edited the manuscript. All authors contributed to the article and approved the submitted version.

## Conflict of Interest

AMB reports an unrestricted grant from Sanofi-Aventis for a study in type 2 diabetes patients on insulin therapy (support of self-management by mHealth), outside the submitted work. GR received an unrestricted research grant from Sanofi Aventis and fees from Novo Nordisk for consultancy and lecturing, outside the submitted work. FC received a fee from Novo Nordisk for a lecture (2016), not related to the submitted work. MA reports an unrestricted grant from Sanofi Aventis for conducting studies (till 2010) regarding insulin therapy in type 2 diabetes patients. RV reports an unrestricted grant from Sanofi-Aventis for a study in type 2 diabetes patients on insulin therapy (support of self-management by mHealth) outside the submitted work.

## References

[1] Sharma M Nazareth I Petersen I. Trends in incidence, prevalence and prescribing in type 2 diabetes mellitus between 2000 and 2013 in primary care: A retrospective cohort study. BMJ Open (2016) 6(1):e010210. 10.1136/bmjopen-2015-010210 PMC4735176 26769791

[2] Wallia A Molitch ME. Insulin therapy for type 2 diabetes mellitus. JAMA - J Am Med Assoc (2014) 311(22):2315–25. 10.1001/jama.2014.5951 24915263

[3] Polonsky WH Fisher L Guzman S Villa-Caballero L Edelman SV. Psychological insulin resistance in patients with type 2 diabetes: The scope of the problem. Diabetes Care (2005) 28(10):2543–5. 10.2337/diacare.28.10.2543 16186296

[4] Hunt LM Valenzuela MA Pugh JA. NIDDM patients’ fears and hopes about insulin therapy. The basis of patient reluctance. Diabetes Care (1997) 20(3):292–8. 10.2337/diacare.20.3.292 9051375

[5] Korytkowski M. When oral agents fail: Practical barriers to starting insulin. Int J Obes (2002) 26:S18–24. 10.1038/sj.ijo.0802173 12174319

[6] Larkin ME Capasso VA Chen CL Mahoney EK Hazard B Cagliero E. Measuring psychological insulin resistance: barriers to insulin use. Diabetes educator (2008) 34(3):511–7. 10.1177/0145721708317869 18535324

[7] Peyrot M Rubin RR Lauritzen T Skovlund SE Snoek FJ Matthews DR. Resistance to insulin therapy among patients and providers: Results of the cross-national Diabetes Attitudes, Wishes, and Needs (DAWN) study. Diabetes Care (2005) 28(11):2673–9. 10.2337/diacare.28.11.2673 16249538

[8] Kunt T Snoek FJ. Barriers to insulin initiation and intensification and how to overcome them. Int J Clin Pract (2009) 63(SUPPL. 164):6–10. 10.1111/j.1742-1241.2009.02176.x 19751453

[9] Yang W Zhuang X Li Y Wang Q Bian R Shen J. Improvements in quality of life associated with biphasic insulin aspart 30 in type 2 diabetes patients in China: results from the A1chieve(R) observational study. Health Qual Life Outcomes (2014) 12(38):137. 10.1186/s12955-014-0137-9 25424627 PMC4253979

[10] Opsteen C Qi Y Zinman B Retnakaran R. Effect of short-term intensive insulin therapy on quality of life in type 2 diabetes. J Eval Clin Pract (2012) 18(2):256–61. 10.1111/j.1365-2753.2010.01552.x 20846320

[11] Houlden R Ross S Harris S Yale JF Sauriol L Gerstein HC. Treatment satisfaction and quality of life using an early insulinization strategy with insulin glargine compared to an adjusted oral therapy in the management of Type 2 diabetes: The Canadian INSIGHT Study. Diabetes Res Clin Practice (2007) 78(2):254–8. 10.1016/j.diabres.2007.03.021 17490781

[12] Braun A Sämann A Kubiak T Zieschang T Kloos C Müller UA. Effects of metabolic control, patient education and initiation of insulin therapy on the quality of life of patients with type 2 diabetes mellitus. Patient Educ Couns (2008) 73(1):50–9. 10.1016/j.pec.2008.05.005 18583087

[13] Goudswaard AN Stolk RP Zuithoff P De Valk HW Rutten GE. Starting insulin in type 2 diabetes: Continue oral hypoglycemic agents? A randomized trial in primary care. J Family Pract (2004) 53(5):393–9. 15125825

[14] Reza M Taylor CD Towse K Ward JD Hendra TJ. Insulin improves well-being for selected elderly type 2 diabetic subjects. Diabetes Res Clin Practice (2002) 55(3):201–7. 10.1016/S0168-8227(01)00327-8 11850096

[15] Yki-Järvinen H Kauppila M Kujansuu E Lahti J Marjanen T Niskanen L. Comparison of insulin regimens in patients with non-insulin-dependent diabetes mellitus. New Engl J Med (1991) 327(20):1426–33. 10.1056/NEJM199211123272005 1406860

[16] Alvarsson M Sundkvist G Lager I Berntorp K Fernqvist-Forbes E Steen L. Effects of insulin vs. glibenclamide in recently diagnosed patients with type 2 diabetes: a 4-year follow-up. Diabetes Obes Metab (2008) 10(5):421–9. 10.1111/j.1463-1326.2007.00719.x 17394534

[17] Venskutonyte L Brismar K Rydén-Bergsten T Rydén L Kjellström B. Satisfaction with glucose-lowering treatment and well-being in patients with type 2 diabetes and myocardial infarction: A DIGAMI2 QoL sub-study. Diabetes Vasc Dis Res (2013) 10(3):263–9. 10.1177/1479164112463711 23188892

[18] Oliveira RA Tostes M Queiroz VA Rodacki M Zajdenverg L. Insulin mediated improvement in glycemic control in elderly with type 2 diabetes mellitus can improve depressive symptoms and does not seem to impair health-related quality of life. Diabetol Metab Syndrome (2015) 7(1):2–7. 10.1186/s13098-015-0052-1 PMC4478689 26110026

[19] de Sonnaville J Snoek F Colly L Devillé W Wijkel D Heine R. Well-being and symptoms in relation to insulin therapy in type 2 diabetes. Diabetes Care (1998) 21(6):919–24. 10.2337/diacare.21.6.919 9614608

[20] de Grauw WJ van de Lisdonk EH van Gerwen WH van den Hoogen HJ van Weel C. Insulin therapy in poorly controlled type 2 diabetic patients: does it affect quality of life? Br J Gen practice: J R Coll Gen Practitioners (2001) 51(468):527–32. PMC1314043 11462311

[21] Lingvay I Legendre J Kaloyanova PF Zhang S Adams-Huet B Raskin P. Insulin-Based Versus Triple Oral Therapy for Newly Diagnosed Type 2 Diabetes. Diabetes Care (2009) 32(10):1789–95. 10.2337/dc09-0653 PMC2752924 19592630

[22] Goddijn PP Bilo HJ Feskens EJ Groeniert KH van der Zee KI Meyboom-de Jong B. Longitudinal study on glycaemic control and quality of life in patients with Type 2 diabetes mellitus referred for intensified control. Diabetic Med (1999) 16(1):23–30. 10.1046/j.1464-5491.1999.00002.x 10229289

[23] Redekop K Koopmanschap M Stolk R Rutten G Wolffenbuttel B Niessen L. Health-Related Quality of Life and Treatment Satisfaction in Dutch Patients With Type 2 Diabetes. Diabetes Care (2002) 25(July):458–63. 10.2337/diacare.25.3.458 11874930

[24] Davis TME Clifford RM Davis WA. Effect of insulin therapy on quality of life in Type 2 diabetes mellitus: The Fremantle Diabetes Study. Diabetes Res Clin Practice (2001) 52(1):63–71. 10.1016/S0168-8227(00)00245-X 11182217

[25] Tamir O Wainstein J Raz I Shemer J Heymann A. Quality of Life and Patient-Perceived Difficulties in the Treatment of Type 2 Diabetes. Rev Diabetic Stud (2012) 9(1):46–54. 10.1900/RDS.2012.9.46 22972444 PMC3448173

[26] Davis TME Bruce DG Curtis BH Barraclough H Davis WA. The relationship between intensification of blood glucose-lowering therapies, health status and quality of life in type 2 diabetes: The Fremantle Diabetes Study Phase II. Diabetes Res Clin Practice (2018) 142:294–302. 10.1016/j.diabres.2018.05.047 29879496

[27] Cleveringa F Gorter K Van Den Donk M Rutten G. Combined Task Delegation, Computerized Decision Support, and Feedback Improve Cardiovascular Risk for Type 2 Diabetic Patients. Diabetes Care (2008) 31(12):2273–5. 10.2337/dc08-0312 PMC2584178 18796619

[28] ATC / DDD Index. WHO Collaborating Centre for Drug Statistics Methodology. (2020). Available at: https://www.whocc.no/atc_ddd_index/.

[29] Aaronson NK Muller M Cohen PDA Essink-Bot ML Fekkes M Sanderman R. Translation, validation, and norming of the Dutch language version of the SF-36 Health Survey in community and chronic disease populations. J Clin Epidemiol (1998) 51(11):1055–68. 10.1016/S0895-4356(98)00097-3 9817123

[30] Goddijn P Bilo H Meadows K Groenier K Feskens E Meyboom-de Jong B. The validity and reliability of the Diabetes Health Profile (DHP) in NIDDM patients referred for insulin therapy. Qual Life Res (1996) 5(4):433–42. 10.1007/BF00449918 8840823

[31] van Buuren S Groothuis-Oudshoorn K. mice: Multivariate imputation by chained equations in R. J Stat Softw (2011) 45(3):1–67. 10.18637/jss.v045.i03

[32] Holm S. A Simple Sequentially Rejective Multiple Test Procedure. Scand J Stat (1979) 6(2):65–70.

[33] Hays RD Morales LS. The RAND-36 measure of health-related quality of life. Ann Med (2001) 33(5):350–7. 10.3109/07853890109002089 11491194

[34] Mulhern B Meadows K. Investigating the minimally important difference of the Diabetes Health Profile (DHP-18) and the EQ-5D and SF-6D in a UK diabetes mellitus population. Health (2013) 05(06):1045–54. 10.4236/health.2013.56140

[35] Pouwer F Hermanns N. Insulin therapy and quality of life. A review. Diabetes/Metab Res Rev (2009) 25:S4–10. 10.1002/dmrr.981 19662621

[36] Billings LK Handelsman Y Heile M Schneider D Wyne K. Health-related quality of life assessments with once-weekly glucagon-like peptide-1 receptor agonists in type 2 diabetes mellitus. J Managed Care Specialty Pharm (2018) 24(9):S30–41. 10.18553/jmcp.2018.24.9-a.s30 PMC10408424 30156447

