# Supplementary material for: Insulin Therapy in Type 2 Diabetes Is Associated With Barriers to Activity and Worse Health Status: A Cross-Sectional Study in Primary Care
Source: Front Endocrinol (Lausanne). 2021 Mar 10;12:573235. doi: 10.3389/fendo.2021.573235 (PMC7989698; doi:10.3389/fendo.2021.573235)
Supplement: Supplementary file 1 [file Table_1.docx]

**Supplementary file 1.** Comparison of the characteristics of individuals with any missing value and those with completely observed data for the outcome (DHP-1 and SF-36).

|  | Individuals with any missing value(s) | Individuals with completely observed data |
| --- | --- | --- |
| n | 1352 | 1442 |
| Age in years, mean (SD) | 66.79 (11.72) | 64.84 (10.36) |
| Women, n (%) | 740 (54.7) | 680 (47.2) |
| Duration of diabetes in years, mean (SD) | 6.1 (6.2) | 5.9 (5.7) |
| Western European ethnicity, n (%) | 1115 (89.4) | 1276 (97.3) |
| Level of education, n (%) |  |  |
| low | 535 (71.9) | 554 (63.9) |
| medium | 170 (22.8) | 219 (25.3) |
| high | 39 (5.2) | 94 (10.8) |
| Insulin use, n (%) | 107 (7.9) | 105 (7.3) |
| Microvascular complication, n (%) | 469 (41.2) | 451 (36.6) |
| Macrovascular complication, n (%) | 243 (18.0) | 247 (17.1) |
| Depression, n (%) | 103 (7.6) | 81 (5.6) |
| BMI, mean (SD) | 30.0 (5.4) | 30.2 (5.3) |
| Systolic blood pressure in mmHg, mean (SD) | 148.62 (22.39) | 149.08 (20.48) |
| HbA1c in %, mean (SD) | 7.2 (1.3) | 7.1 (1.1) |
| LDL-cholesterol in mmol/l, mean (SD) | 2.72 (0.94) | 2.73 (0.90) |

**Supplementary file 2.** Full imputation strategy

| **Imputation method** | Multiple imputation | | |  |
| --- | --- | --- | --- | --- |
| **Number of imputed datasets** | 10 | | **Number of iterations** | 70 |
| **Software used** | R version 1.0.143, “mice” package | | | |
| **Interactions** | No | |  |  |
| **Imputed variable** | **% missing** | **Used in analyses as** | **Method used for imputation** | **Predictors used for imputation** |
| SF-36 Physical functioning | 32.8% | Outcome, continuous | Predictive mean matching | Diabetes duration, age, sex, ethnicity, alcohol use, body mass index, fasting plasma glucose at baseline and after one year, systolic blood pressure, low-density lipoprotein cholesterol at baseline and after one year, high-density lipoprotein cholesterol, cholesterol ratio, triglycerides, myocardial infarction, angina pectoris, cerebrovascular accidents, presence of any diabetes complication, macrovascular complication, microvascular complication, retinopathy, retinopathy classification, neuropathy, nephropathy, Diabetes Empowerment Scale, EuroQol-Five Dimension-Visual Analogue Scale at baseline and after one year, Diabetes Treatment Satisfaction Questionnaire score, insuline use, oral antihyperglycaemic agents use, depression, level of education, Diabetes Health Profile at baseline and after one year, Short Form-36 at baseline and after one year |
| SF-36 Social functioning | 36.2% | Outcome, continuous | Predictive mean matching |  |
| SF-36 Role physical | 34.9% | Outcome, continuous | Predictive mean matching |  |
| SF-36 Role emotional | 36.1% | Outcome, continuous | Predictive mean matching |  |
| SF-36 Mental health | 32.2% | Outcome, continuous | Predictive mean matching |  |
| SF-36 Vitality | 32.9% | Outcome, continuous | Predictive mean matching |  |
| SF-36 Bodily pain | 32.0% | Outcome, continuous | Predictive mean matching |  |
| SF-36 General health | 33.0% | Outcome, continuous | Predictive mean matching |  |
| SF-36 Health change | 33.0% | Outcome, continuous | Predictive mean matching |  |
| DHP Barriers to activity | 36.5% | Outcome, continuous | Predictive mean matching |  |
| DHP Psychological distress | 36.6% | Outcome, continuous | Predictive mean matching |  |
| DHP Disinhibited eating | 33.6% | Outcome, continuous | Predictive mean matching |  |
| Ethnicity | 8.4% | Confounder, categorical | Logistic regression |  |
| Level of eduation | 42.3% | Confounder, ordinal | Polytomous regression |  |
| Duration diabetes (y) | 6.2% | Confounder, continuous | Predictive mean matching |  |
| Microvascular complications | 15.2% | Confounder, binary | Logistic regression |  |
| Body mass index (kg/m^2^) | 0.1% | Confounder, continuous | Predictive mean matching |  |
| Systolic blood pressure (mmHg) | 0.1% | Confounder, continuous | Predictive mean matching |  |
| HbA1c (%) | 1.1% | Confounder, continuous | Predictive mean matching |  |
| LDL cholesterol (mmol/l) | 3.1% | Confounder, continuous | Predictive mean matching |  |

Age, sex, insulin use, macrovascular complications, depression: no missing value
Abbreviations: DHP = Diabetes Health Profile; LDL = low-density lipoprotein; SF-36 = Short Form-36.

**Supplementary file 3.** types of insulin used by the study population

| **Insulin type** | **N** | **%*** |
| --- | --- | --- |
| *Rapid acting* |  |  |
| Lispro | 8 | 3.8 |
| Glulisine | 1 | 0.5 |
| Aspart | 17 | 8 |
| *Short acting* |  |  |
| Regular | 13 | 6.1 |
| *Intermediate acting* |  |  |
| Isophane (NPH) | 25 | 11.8 |
| *Long acting* |  |  |
| Glargine | 37 | 17.5 |
| Detemir | 3 | 1.4 |
| *Pre-mixed* |  |  |
| Isophane/regular | 68 | 32.1 |
| Lispro/lispro protamine | 8 | 3.8 |
| Aspart/apart protamine | 58 | 27.4 |

*percentages do not add up to 100% since patients could use more than one type of insulin
